# Effect of routine iron supplementation with or without folic acid on anemia during pregnancy

**DOI:** 10.1186/1471-2458-11-S3-S21

**Published:** 2011-04-13

**Authors:** Mohammad Yawar Yakoob, Zulfiqar A Bhutta

**Affiliations:** 1 Division of Women & Child Health, The Aga Khan University, Karachi, Pakistan

## Abstract

**Introduction:**

Iron deficiency is the most prevalent nutrient deficiency in the world, particularly during pregnancy. According to the literature, anemia, particularly severe anemia, is associated with increased risk of maternal mortality. It also puts mothers at risk of multiple perinatal complications. Numerous studies in the past have evaluated the impact of supplementation with iron and iron-folate but data regarding the efficacy and quality of evidence of these interventions are lacking. This article aims to address the impact of iron with and without folate supplementation on maternal anemia and provides outcome specific quality according to the Child Health Epidemiology Reference Group (CHERG) guidelines.

**Methods:**

We conducted a systematic review of published randomized and quasi-randomized trials on PubMed and the Cochrane Library as per the CHERG guidelines. The studies selected employed daily supplementation of iron with or without folate compared with no intervention/placebo, and also compared intermittent supplementation with the daily regimen. The studies were abstracted and graded according to study design, limitations, intervention specifics and outcome effects. CHERG rules were then applied to evaluate the impact of these interventions on iron deficiency anemia during pregnancy**.** Recommendations were made for the Lives Saved Tool (LiST).

**Results:**

After screening 3550 titles, 31 studies were selected for assessment using CHERG criteria. Daily iron supplementation resulted in 73% reduction in the incidence of anemia at term (RR = 0.27; 95% CI: 0.17 – 0.42; random effects model) and 67% reduction in iron deficiency anemia at term (RR = 0.33; 95% CI: 0.16 – 0.69; random model) compared to no intervention/placebo. For this intervention, both these outcomes were graded as ‘moderate’ quality evidence. Daily supplementation with iron-folate was associated with 73% reduction in anemia at term (RR = 0.27; 95% CI: 0.12 – 0.56; random model) with a quality grade of ‘moderate’. The effect of the same intervention on iron deficiency anemia was non-significant (RR = 0.43; 95% CI: 0.17 – 1.09; random model) and was graded as ‘low’ quality evidence. There was no difference in rates of anemia at term with intermittent iron-folate vs. daily iron-folate supplementation (RR = 1.61; 95% CI: 0.82 –3.14; random model).

**Conclusion:**

Applying the CHERG rules, we recommend a 73% reduction in anemia at term with daily iron (alone) supplementation or iron/folate (combined) vs. no intervention or placebo; for inclusion in the LiST model. Given the paucity of studies of intermittent iron or iron-folate supplementation, especially in developing countries, we recommend further evaluation of this intervention in comparison with daily supplementation regimen.

## Introduction

Around 2 billion people, amounting to over 30% of the world’s population are anemic, mainly due to iron deficiency [1]. Iron deficiency is the most prevalent and also the most neglected nutrient deficiency in the world, particularly among pregnant women and children, especially in developing countries [2]. It is also significantly prevalent in industrialized countries. Estimates say that globally, fifty six million pregnant women (41.8% of the total) are affected with anemia, again mostly due to iron deficiency [3]. In developing countries, this proportion can be as high as 80% like in South Asia [4], making pregnant mothers susceptible to increased risk of mortality and decreased work capacity. It may also lead to other perinatal complications like pre-eclampsia, low birth weight, prematurity and perinatal mortality [5]. It is the poorest, most vulnerable and least educated who are disproportionately affected by iron deficiency, and it is this group that stands to gain the most by its reduction.

Anemia, as defined by low hemoglobin or hematocrit, is commonly used to assess the severity of iron deficiency in populations without high rates of malaria. The high physiological requirement for iron in pregnancy is difficult to meet with most diets. Therefore, pregnant women should routinely receive iron supplementation, especially in developing countries. Prenatal iron supplementation is not compulsory in many industrialized countries and the recommended dose is usually small (30 mg ferrous iron daily) [6]. However, for developing countries, the recommendation is a daily dose of 60 mg of iron for pregnant, non-anemic women for six months and an increased dose of 120 mg of iron daily if the duration of supplementation is shorter, if iron deficiency prevalence in women of a given country is high, and if pregnant women are anemic. This supplement should include 400 µg of folic acid or lower doses if this amount is not available [7].

Earlier studies have provided sufficient evidence to show that iron supplementation with or without folic acid results in a significant reduction in the incidence of anemia during pregnancy [2,8]. There has also been a limited impact of iron supplementation in community settings owing to lack of compliance and poor infrastructure [9]. However, data regarding quality of evidence for the effectiveness of iron during pregnancy are lacking. Besides, the data on studies in developing countries have not been presented separately. This article is one of the series of papers that aim to determine efficacy of interventions for recommendations into the Lives Saved Tool (LiST), especially in developing countries and is, therefore, different from previously published systematic reviews. In LiST, increases in coverage of an intervention results in a reduction of risk factor or one or more causes of mortality. In this review, the recommendations are made based on application of adapted Grading of Recommendations, Assessment, Development and Evaluation (GRADE) approach for the quality of evidence and use of the Child Health Epidemiology Reference Group (CHERG) rules. For more details of the review methods, the adapted GRADE approach or the LiST model, see the methods paper for by CHERG group [10].

## Methods

### Searching

We systematically reviewed all published literature up to June 21, 2010 to identify studies of iron supplementation with or without folic acid during pregnancy on maternal anemia. As per the Child Health Epidemiology Reference Group (CHERG) systematic review guidelines [10], we searched PubMed and the Cochrane Library, and included publications in any language available in these databases. Every effort was made to gather unpublished data when reports were available for full abstraction. Previous reviews on the topic were also hand-searched to look for relevant studies [2,8,11]. We used the Medical Subject Heading Terms (MeSH) and free text terms for the search strategy using a combination of terms for iron, folic acid and pregnancy, as follows:

("Iron"[Mesh OR "Folic Acid"[Mesh] OR iron OR folic acid OR folate) AND ("Anemia"[Mesh] OR "Anemia, Iron-Deficiency"[Mesh] OR anemia) AND (pregnancy OR maternal)

### Inclusion/exclusion criteria

We limited our search to randomized and quasi-randomized trials conducted in both developed and developing countries, comparing the effects of preventive prenatal oral iron or iron + folic acid supplements among pregnant women versus no treatment/placebo. The developing countries were defined as countries with Gross National Income per capita (GNI) below US$11,905, according to World Bank [12]. Pregnant mothers could be of any age or parity. Studies were included if iron or iron-folate was given alone to the intervention group. Those studies were excluded that assessed the effects of multiple combinations of vitamins and minerals except even if iron/iron-folate was the only difference among the study groups (arms). All included studies contained a placebo or a suitable control group that did not contain iron or iron-folate. There were no limits on gestational age at the time of enrolment in the study and the duration of supplementation. Studies of peri-conceptional or postpartum iron/iron-folate supplementation were excluded. Studies of fortification of iron/iron-folate in food or studies in which iron was given in forms other than oral supplements like powder were excluded. Similarly, studies where iron or iron-folate was given in any parenteral formulation were excluded. Other exclusion criteria included trials of supplementation with multiple micronutrients (MMN) containing iron or iron + folic acid in comparison to supplementation with iron or iron + folic acid alone as this has been reviewed in another paper of this series [13]. Studies where iron/iron-folate was given to anemic pregnant women as a medical treatment were also excluded as the primary objective of this review was to assess the efficacy of preventive iron/folate supplementation for anemia during pregnancy. The therapeutic role of iron for anemia during pregnancy had been reviewed elsewhere [14]. We conducted sub-group analyses with respect to study country setting i.e. developed or developing. However, we did not specifically evaluate minor adverse effects of the supplements such as nausea, vomiting, headache or constipation among the pregnant mothers.

### Abstraction, analyses and summary measures

Studies were included if data from one of the following outcomes was provided: anemia at term, iron deficiency anemia at term, severe anemia at term and severe anemia at any time during the second and third trimester. All outcome measures to be included were determined a priori. The interventions described in this review can be subdivided into four categories: 1) daily iron supplementation alone compared to placebo/control, 2) weekly iron supplementation alone compared to daily regimen, 3) daily supplementation of iron and folic acid versus placebo/control and 4) weekly supplementation of iron and folic acid versus daily supplementation.

All studies that met final inclusion and exclusion criteria were double-data abstracted into a standardized form for each outcome of interest. We abstracted key variables with regard to the study identifiers and context, study design and limitations, intervention specifics, and outcome effects. Each study was assessed and graded according to the CHERG adaptation of the GRADE technique [15]. Studies received an initial score of high if they were RCTs or cluster-RCTs (cRCTs). The grade was decreased by 0.5 - 1 point for each study design limitation like inadequate methods of sequence generation, allocation concealment and attrition > 20% etc. In addition, studies reporting an intent-to-treat analysis or with statistically significant strong levels of association (> 80% reduction) received 0.5 – 1.0 grade increase. Any study with a final grade of very low was excluded on the basis of inadequate study quality.

For any outcome with more than one study, we conducted a meta-analysis and reported the Mantel-Haenszel pooled relative risk and corresponding 95% confidence interval (CI). In case of heterogeneity (P < 0.1 and I^2^ > 50%), the random effect model (DerSimonian-Laird) pooled relative risk and corresponding 95% CI was used, especially where there was unexplained heterogeneity such as major differences in study design [10]. All analyses were conducted using RevMan 5 statistical software.

We summarized the evidence based on outcome by including assessment of the study quality and quantitative measures according to standard guidelines [10] for each outcome. For the outcomes of interest, namely the effect of iron/iron-folate on maternal anemia, we applied the CHERG Rules for Evidence Review [10] to recommend final estimates for reduction in anemia with iron or iron-folate supplementation. Additional file 1 contains a list of studies from the search that were excluded from the meta-analyses with a brief explanation for why the study was excluded.

### Definitions

Anemia was defined as hemoglobin (Hb) level of less than 110g/L and severe anemia was defined as hemoglobin level of less than 70g/L. Iron deficiency anemia was defined as Hb less than 110 g/L and at least one additional laboratory indicator (mean cell volume, haemoglobin concentration, serum ferritin, erythrocyte protoporphyrin concentrations etc) of iron deficiency .

## Results

**Figure 1:**
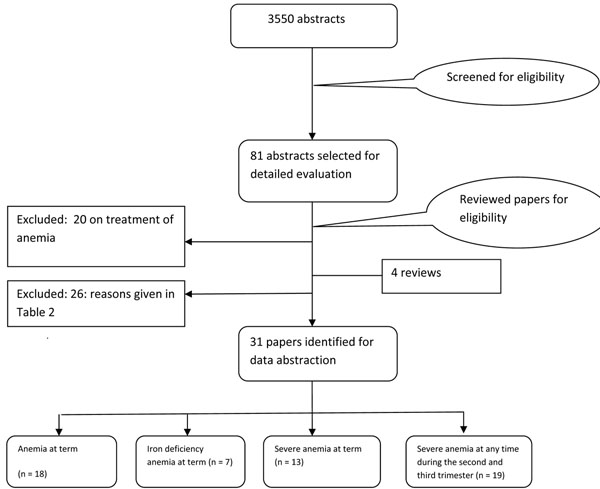
Flow chart of the literature search

The search generated 3550 hits on PubMed and 290 in Cochrane Library that were screened and after removing duplicates, 81 abstracts were preliminarily selected. These were reviewed in detail, including full texts and finally 31 [16-47] studies were selected for inclusion in this paper. A detailed account of the number of abstracts and titles scanned is given as a flow chart (Figure 1). Table 1 gives the summary of the quality of evidence and the impact estimates of different interventions.

**Table 1:** Quality assessment of trials of iron and/or folate on the incidence of anemia during pregnancy

	Quality Assessment					Summary of Findings		
**No of studies (ref)**	**Design**	**Limitations**	**Consistency**	**Directness**		**No of events**		
				
				**Generalizability to population of interest**	**Generalizability to intervention of interest**	**Intervention**	**Control**	**Relative Risk (95% CI)**

**Daily iron versus no intervention/placebo *Anemia at term: Moderate outcome specific quality***								

14	RCT/quasi RCT	Studies with unclear or inadequate sequence generation and high loss to follow-up	All studies show direction of benefit, but high heterogeneity	Only two studies in developing countries, rest in developed nations	Yes	114	313	RR (random) = 0.27 [ 0.17, 0.42 ]

**Daily iron versus no intervention/placebo*: Iron deficiency anemia at term: Moderate outcome specific quality***								

6	RCT/quasi RCT	Studies with unclear or inadequate sequence generation and high loss to follow-up	All studies show direction of benefit, with borderline heterogeneity	Only one study in developing country, rest in developed	Yes	25	68	RR (random) = 0.33 [ 0.16, 0.69 ]

**Daily iron and folic acid versus no intervention/placebo*: Anemia at term: Moderate outcome specific quality***								

3	RCT/quasi-RCTs	High loss to follow-up and unclear sequence generation	Two studies show direction of benefit; while the third study had zero events	Only one study in developing country, rest in developed	Yes	17	49	RR (random) = 0.27 [ 0.12, 0.56 ]

**Daily iron and folic acid versus no intervention/placebo: *Iron deficiency anemia at term: Low outcome specific quality***								

1	RCT	Allocation concealment unclear and blinding inadequate	One study only	In a developed country	Yes	12	5	RR (random) = 0.43 [0.17, 1.09]

## Anemia at term

**Figure 2:**
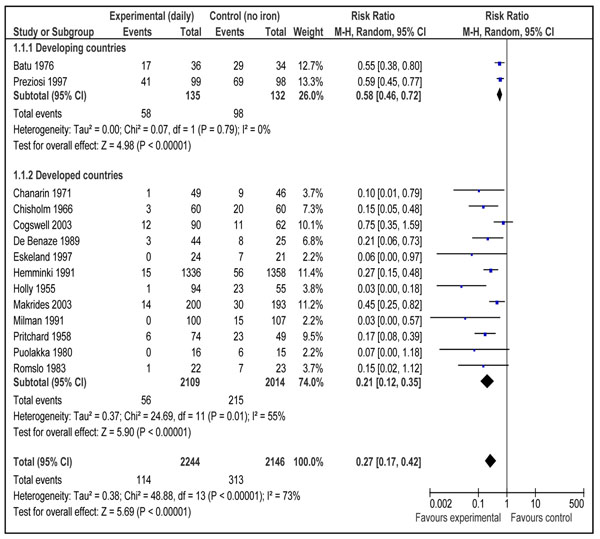
Impact of daily iron supplementation compared with no supplementation on anemia at term (hemoglobin less than 110g/L)

**Figure 3:**
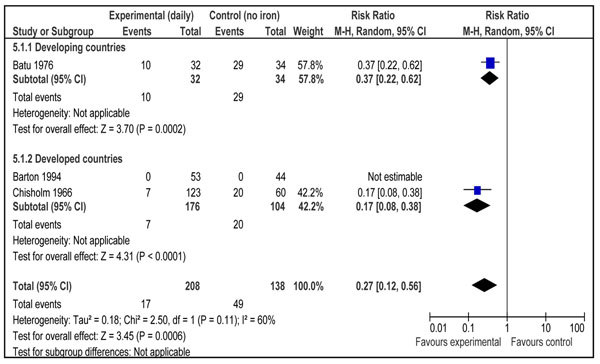
Impact of daily supplementation with iron and folate compared with no supplementation on anemia at term (hemoglobin less than 110g/L)

**Figure 4:**
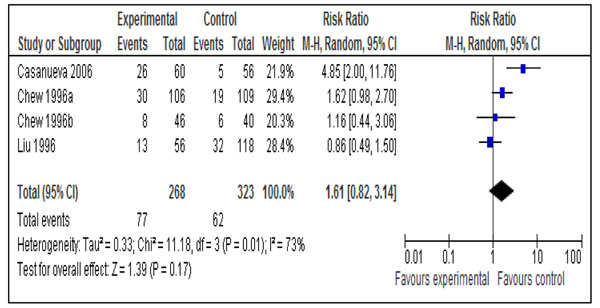
Impact of intermittent supplementation with iron and folic acid compared with daily supplementation on anemia at term (hemoglobin less than 110g/L)

This outcome was reported by 18 studies [16,17,19-21,23,24,27,29,30,32-34,37-39,41,47]. Daily supplementation with iron only versus no intervention/placebo was evaluated by 14 studies [17,19,21,23,24,27,29,30,33,34,37-39,41]. There was a statistically significant 73% reduction in the incidence of anemia at term (RR = 0.27; 95% CI: 0.17 – 0.42; random model) (Figure 2). There were three studies [16,17,21] that evaluated daily supplementation with iron and folic acid both versus no intervention/placebo and pooled data also showed a significant 73% reduction in anemia at term (RR = 0.27; 95% CI: 0.12 – 0.56; random model) (Figure 3). There was, however, no difference between intermittent iron-folic acid supplementation vs. daily iron-folate on this outcome based on three studies [20,32,47] (RR = 1.61; 95% CI: 0.82 –3.14; random model) (Figure 4).

## Iron deficiency anemia at term

This outcome was reported by seven studies [23,27,31,33,34,43,45]. Daily supplementation with iron alone versus no intervention/placebo had a significant 67% reduction in incidence of iron deficiency anemia at term based on six studies [23,27,33,34,43,45] (RR = 0.33; 95% CI: 0.16 – 0.69; random model). There was one study [31] on daily iron-folic acid versus no intervention/placebo and the effect was large but non-significant (RR = 0.43; 95% CI: 0.17 – 1.09; random model)

## Severe anemia at term

This outcome was estimated by 13 studies [16,18,20,23,27,30-35,41,45]. Daily iron supplementation versus no intervention/placebo, as seen in 8 studies [18,23,27,30,33,34,41,45], had a non significant adverse impact on severe anemia at term (RR = 4.83; 95% CI: 0.23 - 99.88; random model). This result was primarily based on one study [33] as all the other studies had zero events in both groups. Three studies evaluated the impact of daily supplementation with iron and folate both on severe anemia at term [16,18,31], but the number of events in these studies in both groups was nil. Intermittent supplementation with iron and folate vs. daily supplementation of same micronutrients also yielded no severely anemic patients in the intervention and control groups in the three studies that looked at this outcome [20,32,35].

## Severe anemia at any time during second and third trimester

Severe anemia during second and third trimester was reported by 19 studies [16,18,22,23,25,27,28,30-36,40,42,44-46]. Daily iron alone compared to no supplementation had no impact on severe anemia in second and third trimester based on nine studies [18,22,23,27,28,30,33,34,45], (RR = 0.48; 95% CI: 0.01 – 34.52; random model), with only two studies [22,33] having events greater than zero in the intervention and control groups. For intermittent iron alone vs. daily iron alone, there were two studies [36,44] that looked at this outcome and both had zero events in the two groups. Daily iron-folic acid versus no intervention/placebo also had no effect on severe anemia at any time during second and third trimester based on four studies [16,18,22,31] (RR = 0.11; 95% CI: 0.01 – 0.83), with zero events in three of these studies in both groups, except one [22]. Intermittent iron-folate vs. daily iron-folate outcome included 6 studies [25,32,35,40,42,46] and events were nil in both groups in all these six studies.

## Discussion

Iron supplementation alone or in combination with folic acid has been associated with the well being of the mother and fetus. It leads to a significant reduction in anemia incidence during pregnancy and, thus, plays a vital role in reducing maternal morbidity and mortality. The results of our review were consistent with those of an earlier Cochrane review by Pena Rosas and Viteri [2] that also showed a significant reduction in incidence of anemia and iron deficiency anemia at term with daily iron supplementation and that of anemia at term due to daily iron-folate supplementation versus no intervention/placebo.

The pooled analysis of effect of daily iron supplementation vs. control had a high heterogeneity (fig 2). The most likely explanation of this substantial statistical heterogeneity (I^2^ =73 %) is the variable effect size of the studies which in turn depend on the baseline anemia status of the study population. An important observation to make is that the direction effect in all the studies was in the same direction. We can expect that biologic effect of iron supplementation would differ based on prevalence of anemia in the study population. To further elaborate on this observation, we conducted a post hoc subgroup analysis based on baseline anemia status of the study population (data not shown). There were seven studies that included only non-anemic pregnant women based on laboratory evidence of absence of anemia (hemoglobin < 110 g/L) [21,23,24,27,29,33,39] and in other seven studies, population was that of mixed status [17,19,30,34,37,38,41]. Pooled estimates for non-anemic women were less heterogeneous (I^2^ =47%) and size of summary estimate was less prominent (RR 0.31, 95 % CI 0.19-0.52) compared to that of mixed population that had more heterogeneous (I^2^=83%) and more prominent results (RR 0.22, 95 % CI 0.11-0.47). This shows a strong biologic effect in favor of the intervention and also indicates that effect of Iron supplementation would depend on degree of baseline anemia in the study population.

CHERG rules were applied to the collective outcomes of anemia for recommendation of iron deficiency anemia into the LiST model. Daily supplementation with iron led to 73% reduction in incidence of anemia at term as compared to no supplementation. This intervention had a ‘moderate’ quality evidence owing to some limitations in included studies like unclear [17,21,24,30,34,38,39,41] or inadequate sequence generation [19], and high loss to follow up [17,23,27]. Another limitation was that all the studies in the pooled analysis were not conducted in developing countries. In any case, based on strong biologic plausibility and consistent direction of effect across the studies, we recommend a 73 % reduction in anemia at term with iron supplementation during pregnancy, for inclusion in the LiST model. Daily supplementation with iron and folate led to 73% reduction in incidence of anemia at term when compared with no supplementation. We recommend this estimate for reduction in anemia at term for inclusion in the LiST model. The quality of evidence regarding this intervention had to be down-graded from ‘high’ to moderate due to some limitations like high loss to follow up [17] and unclear sequence generation [21] and also the fact that all the studies were not conducted in developing countries.

Our results show that there was not much difference in effect between iron alone and iron-folate combined. The effect sizes were similar for both the analyses but CIs were wider for that of iron/folate, mainly due to less number of studies in the pooled analysis. This shows that we can expect a similar biological effect when the iron is supplemented alone or in combination with folate. We did a subgroup for developing and developed countries. There were not sufficient studies from developing countries for all the analyses. An important observation to make in figure 2 is that even though there were two studies from developing countries, the results were very consistent (I^2^=0%). Even though there are not a lot of randomized trials conducted in developing countries but we can suggest that iron supplementation would be much effective in developing world by looking at biological plausibility of the intervention. The populations that are most at risk for iron deficiency and IDA are young children and women of reproductive age, especially during pregnancy. The global estimates on prevalence of anemia showed that more than half the pregnant women and young children are anemic in Southeast Asia, West Pacific and Africa [48]. This shows that amount of effect would be substantially high in developing countries however more efficacy trials are required to determine the conduct of the intervention.

Weekly iron and folic acid supplementation (WIFS) is a relatively new phenomenon, and there is very little data at the moment comparing weekly supplementation with daily dosage. Weekly iron and folic acid supplementation, in synchrony with the turnover of mucosal cells, may be a promising substitute for daily iron supplementation and has been proposed as a more efficient preventive approach in public health programs [49,50]. The WHO says that WIFS should be considered a strategy for prevention of iron deficiency in population groups where the prevalence of anemia is above 20% among women of reproductive age and mass fortification programs of staple foods with iron and folic acid are unlikely to be implemented within 1-2 years. However, conclusive data regarding its efficacy as compared to the daily regimen are not yet available. Weekly dosage may have benefits of reduced side effects and increased compliance, but more field randomized controlled trials are needed to establish the efficacy of weekly supplementation compared to daily regimen.

## Conclusions

Iron supplementation has a significant benefit in reducing anemia and iron deficiency anemia at term. Iron in combination with folic acid also has a beneficial impact on anemia at term and should be routinely used in pregnant women at least in developing countries to reduce the incidence of anemia due to increased demands during pregnancy.

## Competing interests

We do not have any financial or non-financial competing interests for this review.

## Authors' contributions

Professor Zulfiqar A Bhutta developed the parameters and scope for the review and secured support. Dr Mohammad Yawar Yakoob undertook the literature search, data extraction and wrote the manuscript along under the supervision of Professor Bhutta.

## Supplementary Material

Additional File 1 Excluded Studies and Reasons for Exclusion. This is an excel file. Click here for file
